# *Harpagophytum procumbens* Inhibits Iron Overload-Induced Oxidative Stress through Activation of *Nrf2* Signaling in a Rat Model of Lumbar Spinal Stenosis

**DOI:** 10.1155/2022/3472443

**Published:** 2022-09-14

**Authors:** Jin Young Hong, Hyunseong Kim, Junseon Lee, Wan-Jin Jeon, Yoon Jae Lee, In-Hyuk Ha

**Affiliations:** Jaseng Spine and Joint Research Institute, Jaseng Medical Foundation, Seoul 135-896, Republic of Korea

## Abstract

Lumbar spinal stenosis (LSS) is a common degenerative spinal condition in older individuals that causes impaired walking and other disabilities due to severe lower back and leg pain. Ligamentum flavum hypertrophy is a major LSS cause that may result from oxidative stress caused by degenerative cascades, including imbalanced iron homeostasis that leads to excessive reactive oxygen species production. We investigated the effects of *Harpagophytum procumbens* (HP) on iron-induced oxidative stress associated with LSS pathophysiology. Primary spinal cord neuron cultures were incubated in FeSO_4_-containing medium, followed by addition of 50, 100, or 200 *μ*g/mL HP. Cell viability was assessed by CCK-8 and live/dead cell assays and by propidium iodide-live imaging. In an *in vivo* rat model of LSS, HP were administered at 100, 200, and 400 mg/kg, and disease progression was monitored for up to 3 weeks. We investigated the *in vitro* and *in vivo* effects of HP on iron-induced neurotoxicity by immunochemistry, real-time PCR, and flow cytometry. HP exerted neuroprotective effects and enhanced neurite outgrowths of iron-injured rat primary spinal cord neurons *in vitro*. HP treatment significantly reduced necrotic cell death and improved cells' antioxidative capacity via the NRF2 signaling pathway in iron-treated neurons. At 1 week after HP administration in LSS rats, the inflammatory response and oxidative stress markers were substantially reduced through regulation of excess iron accumulation. Iron that accumulated in the spinal cord underneath the implanted silicone was also regulated by HP administration via NRF2 signaling pathway activation. HP-treated LSS rats showed gradually reduced mechanical allodynia and amelioration of impaired behavior for 3 weeks. We demonstrated that HP administration can maintain iron homeostasis within neurons via activation of NRF2 signaling and can consequently facilitate functional recovery by regulating iron-induced oxidative stress. This fundamentally new strategy holds promise for LSS treatment.

## 1. Introduction

Lumbar spinal stenosis (LSS) is the most common degenerative spinal condition in older individuals. With the aging of society, the incidence of age-related spinal stenosis has increased [1]. LSS involves ligamentum flavum (LF) hypertrophy, which induces spinal canal narrowing, placing pressure on the spinal cord or nerves and thereby inducing lower back or leg pain [2]. Under normal physiological conditions, the LF provides strong support for the vertebrae and prevents excessive movements of the lower back [3, 4]. However, its function deteriorates with age, accompanied by hypertrophy and fibrosis. There is increasing evidence that aging and age-related diseases are correlated with an oxidative stress condition [5, 6]. Aging is the main risk factor for neurodegenerative disease and involves the decline of cell function and regulation processes, resulting in a decreased antioxidant capacity for cellular homeostasis [7, 8]. Recent studies have reported that oxidative stress mediates LF hypertrophy by inducing inflammation, fibrosis, and apoptosis via Akt and MAPK pathways in LSS patients [9]. In addition, oxidative damage to cellular proteins and organelles was closely related to their restoration or degradation involving homeostatic antioxidative systems [10]. Following the decline in their capacity, oxidatively damaged proteins and organelles can accumulate, leading to degenerative diseases such as degenerative disc disease, Alzheimer's disease, Parkinson's disease, and macular degeneration [11]. Although oxidative stress is believed to be involved in LSS pathological conditions, little is known about the oxidative mechanisms and therapeutic targets for maintaining cellular homeostasis from oxidative stress in spinal stenosis.

Recent evidence has suggested that excessive accumulation of iron over a prolonged period promotes immune cell activation, causing inflammatory responses and ROS production, ultimately leading to an environment of oxidative stress [12–15]. Further, antioxidant capacity is affected by the iron oxidation–reduction (redox) cycle system [16, 17]. Iron is an essential trace element involved in oxygen transport by red blood cells, energy production, DNA synthesis, and cellular respiration [18]. It is also an essential cofactor for key biological activities and biochemical reactions. Insufficient iron levels are known to cause a deficiency of essential iron-containing proteins in the body resulting in diseases, such as iron deficiency anemia, whereas excess iron acts as a catalyst for chemical reactions that produce ROS, leading to oxidative damage in tissues and organs [19]. The expression of regulatory proteins related to iron metabolism is significantly reduced in an injured spinal cord. In addition, the dysregulation of iron homeostasis can ultimately induce oxidative stress and neuronal apoptosis [12]. In another study, iron chelators and oxidative stress inhibitors could effectively relieve spinal cord injury-associated oxidative stress through the nitric oxide synthase–iron-responsive element and iron-regulatory protein pathway [20]. Based on the previous studies, the modulation of iron hemostasis may effectively alleviate neuronal injury via the oxidative stress signaling pathway, resulting in the prevention of secondary damage and eventually leading to functional recovery and pain relief. In the present study, we investigated the role of iron and changes in its metabolism in relation to the oxidative damage caused by LSS. Furthermore, *Harpagophytum procumbens* (HP), clinically used in the treatment of spinal stenosis and the main herbal ingredient used in Jaseng Shinbaro3 and Chungpajeon-H, was administered to an animal model of spinal stenosis to examine its inhibitory effects on oxidative damage and its relationship with the regulation of iron homeostasis. Traditionally, HP extracts are considered as promising herbal medicine in many countries for treating a variety of the diseases, such as intervertebral disc disease, indigestion, osteoarthritis, allergies, and rheumatism, among others [21]. HP has been applied as a traditional medical treatment for lower back pain with spinal stenosis, and its efficacy and safety has been demonstrated [22, 23]. However, the molecular mechanism for the therapeutic effects of HP on LSS has not yet been elucidated. Recent studies have reported that several genes involved in iron homeostasis and metabolism are transcription targets, which was controlled by NRF2 [24]. NRF2-mediated regulation can reduce intracellular labile iron pool to restore homeostasis and prevent oxidative stress in iron overload situations [25]. Thus, we hypothesized that HP inhibits iron overload-induced oxidative stress through activation of NRF2 signaling in LSS. To the best of our knowledge, this is the first study demonstrating the in vitro and in vivo therapeutic effect of HP in LSS. Our results suggest HP treatment to be a potential new strategy for the treatment of LSS via iron metabolism modulation.

## 2. Materials and Methods

### 2.1. Primary Rat Spinal Cord Neuron Cultures

Primary spinal cord neurons were prepared from Sprague–Dawley (SD) rat embryos (Daehan Bio Link, Chungju, Korea) at the age of 15 embryonic days. Briefly, embryos were promptly separated via cesarean section from pregnant rat and placed in petri dishes containing cold Leibovitz's L-15 medium (Gibco-BRL, Grand Island, NY, USA). Embryonic body was positioned with the abdomen facing up, and the spinal cord was carefully isolated by removing the immature spines. Under magnification, dorsal root ganglia (DRG) and meninges were removed from the isolated spinal cord by using fine forceps. The spinal cords were rinsed once in L-15 medium and were then enzymatically digested using the Neural Tissue Dissociation Kit and the gentleMACS™ Dissociator (both Miltenyi Biotec, Bergisch Gladbach, Germany) for 20 min at 37°C. After dissociation, supernatants were discarded after centrifugation at 2,000 rpm for 3 min. The cell pellet was resuspended in 1 mL neurobasal medium supplemented with B27, GlutaMAX, 1% penicillin/streptomycin (all Gibco-BRL), and 10 ng/mL recombinant BDNF (PeproTech, Rocky Hill, CT, USA). Single cells were then seeded onto coated plates with 20 mg/mL poly-D-lysine overnight and with 10 mg/mL laminin (both Gibco-BRL) for 2 h at 4°C.

### 2.2. Preparation of HP Extracts

The HP extract was prepared according to a previously described method [26]: 300 g of HP was heated to 105°C in 3 L of distilled water for 3 h. After cooling at −20°C for 30 min, the HP extract was passed through a filter paper (HA-030, Hyundai Micro, Seoul, Korea) and then lyophilized in a freeze dryer (Ilshin BioBase, Gyeonggi-do, Korea) at −70°C to obtain the dry HP extract. The extract yield was calculated and redissolved in phosphate-buffered saline (PBS; Gibco-BRL) at the desired concentration, after which it was stored at −20°C until use.

### 2.3. Iron(II)Sulfate Heptahydrate-Induced Neuronal Injury and HP Treatment

FeSO_4_ was applied to the spinal cord neurons after cell stabilization for 24 h, as previously described [27]. An iron stock solution was prepared using 27.8 mg FeSO_4_ (Sigma-Aldrich, Oakville, Canada) dissolved in 1 mL PBS. FeSO_4_ was added firstly to the cell culture media at a final concentration of 50 *μ*M for 30 min, followed by the addition of 50, 100, or 200 *μ*g/mL HP extract. The cells were further incubated with both FeSO_4_ and HP extract for 24 h, after which samples were used for the indicated analyses.

### 2.4. Neuronal Viability Assays

The neuronal viability was assessed using Cell Counting Kit-8 assay (CCK-8; Dojindo, Kumamoto, Japan) with 2-fold-increased HP concentrations, without or with FeSO_4_ exposure. After incubation for 24 h, cells in each well were incubated with CCK-8 solution in 10% of total volume for 4 h. Absorbance was then read at 450 nm using an Epoch microplate reader (BioTek, Winooski, VT, USA). Cell viability was calculated as the percentage of surviving neurons relative to the absorbance value of the control cells, set as 100% viability.

Cell viability was also evaluated by means of a live/dead cell imaging kit (Invitrogen, Grand Island, NY, USA). The medium was exchanged to DMEM (Hyclone, Logan, UT, USA) containing a dye solution, and cells were incubated at 37°C for 15 min. The cells were mounted with a fluorescence mounting medium (Dako Cytomation, Carpinteria, CA, USA). Images were randomly captured at 100× magnification with a confocal microscope (Eclipse C2 Plus, Nikon, Minato City, Tokyo, Japan). Live and dead cells were quantified by counting the number of green-stained live cells using ImageJ software (1.37 v, National Institutes of Health, Bethesda, MD, USA).

### 2.5. Immunocytochemistry

Cells were fixed with 4% paraformaldehyde (PFA; Biosesang, Seongnam, Korea) for 30 min and were rinsed three times for 5 min each time with PBS (Gibco-BRL). The cells were permeabilized with 0.2% Triton X-100 in PBS for 5 min, rinsed twice with PBS, and then blocked with 2% normal goat serum (NGS) in PBS for 1 h. The following primary antibodies, rabbit anti-Tuj1 (BioLegend, San Diego, CA, USA, 1 : 2000), rabbit anti-Ferritin (Invitrogen, 1 : 200), rabbit anti-NRF2 (Abcam, Cambridge, UK, 1 : 200), and mouse anti-TFR (Invitrogen, 1 : 1000), were diluted in 2% NGS and treated for overnight at 4°C. Cells were incubated for 2 h with rhodamine phalloidin (F-actin; Invitrogen, 1 : 1000), fluorescein isothiocyanate, or rhodamine-conjugated secondary antibodies (Jackson ImmunoResearch Laboratories, West Grove, PA, USA), diluted at 1 : 300 in 2% NGS. The cells were washed three times for 5 min with PBS, mounted with fluorescence mounting medium (Dako Cytomation), and images were acquired by confocal microscopy (Eclipse C2 Plus; Nikon). Ten representative images were captured at 100× or 400× magnification, using the same acquisition settings. The difference in percentage of ferritin-, TFR-, or NRF2-positive neurons in each group was determined by counting the number of cells labeled positively and was calculated by dividing the number of positive cells by the number of Tuj1^+^ neurons counted per field.

### 2.6. Live-Cell Imaging

The live cell-permeant Hoechst 33342 (0.5 *μ*M/mL, Invitrogen) and the live cell-impermeable propidium iodide (PI; 4 *μ*M/mL, Invitrogen) were added in serum free neurobasal medium. Live-cell imaging was performed every 30 min for 48 h at five sites per well using Tokai Hit, STX series (Controller: STXG, chamber: WSKMX) with a confocal microscope under stage temperature at 37°C, top at 45.5°C, and bath at 41°C in CO_2_ 5%.

### 2.7. Rat LSS Model and HP Administration

All procedures were approved by the Jaseng Animal Care and Use Committee (JSR-2019-09-002-001-A). Male SD rats (7 weeks old, 230–250 g) were obtained from Daehan Bio Link. Rats were housed under constant environment, with free access to food and water. Surgical procedures have been previously described in detail [28]. Briefly, animals were anesthetized with 2–3% isoflurane gas (Forane; BK Pham, Goyang, Korea), and a dorsal laminectomy was performed at L5 using fine rongeur. Next, a silicone block (80 kPa, 4 × 1 × 1 mm^3^) was implanted at the L4 level using fine forceps. Sham-operated rats underwent laminectomy at the L5 level, with no implants. HP extracts were prepared in sterile distilled water immediately before use in a volume of 400 *μ*L and were orally administered in doses of 100, 200, or 400 mg/kg once a day, beginning 30 min after LSS induction and continuing for 1 or 3 weeks after LSS induction, until the animals were sacrificed. The control group received the same volume of distilled water orally.

### 2.8. Histology

Rats were transcardially perfused with 0.9% normal saline (Sigma-Aldrich) and 4% PFA (Biosesang) for histological staining and immunohistochemistry. The lumbar spine containing the L4 segment (i.e., the silicone implantation site) was separated and then postfixed overnight in 4% PFA at 4°C. Spine specimens were cryoprotected in 30% sucrose for 3 days. The samples were sectioned in the sagittal plane at 20-*μ*m thickness. Hematoxylin and eosin (H&E) staining was performed at 3 weeks to evaluate the extent of damage to the spinal cord caused by implantation of the silicone block, according to the standard protocol. The sections were rinsed with PBS, counterstained with hematoxylin solution (Thermo Fisher Scientific) for 2 min, and then rinsed with distilled water. After washing with running tap water for 2 min, the sections were immersed in eosin solution (BBC Biochemical, Mount Vernon, WA, USA) for 10 s and were dehydrated in a graded ethanol series, cleared with xylene, and mounted with VectaMount® Permanent Mounting Medium (Vector, Burlingame, CA, USA). Images of the stained sections were captured with an inverted microscope (Nikon).

### 2.9. Immunohistochemistry

Immunohistochemistry was used to analyze inflammation-, pain-, iron-, and oxidative stress-related protein expression within the spinal cord at the silicone implantation site (L4 level). Sections were incubated with primary antibodies against rabbit anti-CD68 (Abcam, 1 : 500), rabbit anti-TRPV1 (Alomone, Hadassah Ein Kerem, Israel, 1 : 100), guinea pig anti-NeuN (Synaptic Systems, Gottingen, Germany, 1 : 500), mouse antiferritin heavy chain (Santa Cruz Biotechnology, Santa Cruz, CA, USA, 1 : 400), mouse anti-iNOS (R&D Systems, Minneapolis, MN, USA, 1 : 100), rabbit anti-NRF2 (Abcam, 1 : 200), and mouse anti-NF200 (Millipore, Billerica, MA, USA, 1 : 200) overnight at 4°C. After sections were washed three times, secondary antibodies (FITC-coupled goat antimouse or antirabbit or rhodamine-conjugated goat antiguinea pig, Jackson ImmunoResearch Laboratories) were diluted to 1 : 300 in 2% NGS in PBS. Following 2 h of incubation, the sections were washed three times with PBS and mounted with fluorescence mounting medium (DAKO). The stained tissue sections were imaged using confocal microscopy (Eclipse C2 Plus).

The inflammatory response was quantified by manually counting the number of CD68^+^ cells. Transient receptor potential vanilloid subtype 1 (TRPV1)^+^ neurons were also manually counted, and the result was presented as a percentage or ratio. Quantification of neurofilament 200 (NF200)-labeled axons were performed by using ImageJ software. Briefly, NF200-labeled axons were captured at the implantation site under 100× magnification using confocal microscope, and the number of pixels occupied by the NF200 fibers from six images were quantified by dividing the number of NF200^+^ pixels by the number of pixels in ImageJ.

### 2.10. RNA Isolation and Real-Time PCR

Total RNA was isolated from the L4 spinal cord using a RNeasy Mini Kit (Qiagen, Hilden, Germany). cDNA was synthesized using oligo dT primers and AccuPower RT PreMix (Bioneer, Daejeon, Korea). Quantitative real-time PCR (qRT-PCR) was performed in triplicate using the iQ SYBR Green Supermix with a CFX Connect Real-Time PCR Detection System (both Bio-Rad, Hercules, CA, USA), and gene sequences are listed in Table 1. Target gene expression was normalized to that of the *β-actin* and was expressed as the fold-change relative to the control group.

### 2.11. Flow Cytometry

A flow cytometric assay was used to assess apoptosis, using an Annexin V-PE/PI apoptosis detection kit (Abcam) as previously described [29]. Briefly, cells were collected and incubated with 5 *μ*L of Annexin V-PE and 5 *μ*L of PI in 500 *μ*L of 1× binding buffer and directly analyzed by fluorescence-activated cell sorting (FACS; Accuri C6 Plus Flow Cytometer, BD Biosciences, Franklin Lakes, CA, USA). The mean positive cell values, as determined via flow cytometry, were expressed as the percentage relative to the control group.

### 2.12. Enzyme-Linked Immunosorbent Assays

The expression levels of the pro- and anti-inflammatory markers interleukin (IL)-6 and IL-10 in a 1 cm portion of the spinal cord containing the implantation site were evaluated using enzyme-linked immunosorbent assays (ELISAs). Segments were homogenized using a taco™ Prep Bead Beater (GeneReach, Taichung, Taiwan) and centrifuged at 1000 rpm at 4°C for 3 min. Supernatants were examined using ELISA kits (BD Biosciences) following the manufacturer's instructions.

### 2.13. Functional Assessments

We used three tests to assess functional recovery after inducing stenosis. The Basso, Beattie, and Bresnahan (BBB) scale was performed as previously described [26]. Two independent observers analyzed the hindlimb motion in an open field for 4 min. The average value was used for analysis. The ladder walking test was used to test the ability of rats to maintain balance. Rats walked on a metal runway (2.5 cm between grids) from left to right three times, and their movements were recorded with a digital camera. The score was calculated as follows: ladder score (%) = erroneous steps of the hind limb/total steps of the hind limb × 100 [26]. Locomotor functions were examined in each group every 7 days until sacrifice. The Von Frey test was used to measure the response of the rats to pain. We measured the latencies of paw withdrawals in response to mechanical stimulation applied to the center of both hind paws using a Von Frey filament (Ugo Basile, Varese, Italy). The average value from three or more measurements was used. All locomotor tests were recorded using a digital camera and were assessed by two observers who were blinded to the treatment conditions.

### 2.14. Statistical Analyses

All results are expressed as the mean ± standard error of the mean (SEM). Comparisons among each group were analyzed using one-way analysis of variance (ANOVA) with Tukey's post hoc analysis (GraphPad Prism, GraphPad, Inc., La Jolla, CA, USA). Differences were considered as statistically significant if the *p* values <0.05 were considered significant.

## 3. Results

### 3.1. HP Attenuates FeSO_4_-Induced Neurotoxicity in Primary Rat Spinal Cord Neurons

**Figure 1:**
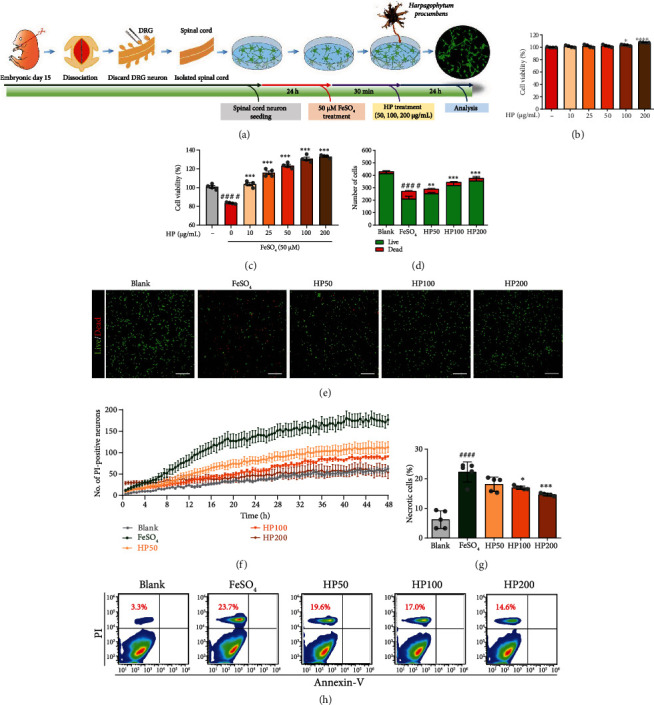
*Harpagophytum procumbens* (HP) attenuates FeSO_4_-induced neurotoxicity in primary spinal cord neurons. (a) Schematic illustration of the experimental protocol of primary spinal cord neurons with HP posttreatment. (b and c) CCK-8 results of spinal cord neurons treated with various concentrations of HP for 24 h, without and with exposure to 50 *μ*M FeSO_4_. (d) Quantification of the number of live and dead cells for the neurotoxicity assay. (e) Cell viability was imaged with confocal microscopy using a live/dead imaging assay kit (live cells, green; dead cells, red). White scale bar = 200 *μ*m. (f) The percentage of PI-positive neurons from live cell imaging over a 48 h period, with images taken every 30 min. (g) Flow cytometric quantification of necrotic cells (Annexin V-negative/PI-positive), challenged with 50 *μ*M FeSO_4_. (h) Representative flow cytometric dot plots showing Annexin V (*x*-axis) and PI (*y*-axis) analysis for cell death type in spinal cord neurons. Data are expressed as the means ± SEM. Significant differences are indicated as ^####^*p* < 0.0001 vs. the blank group, ^∗^*p* < 0.05, ^∗∗^*p* < 0.01, ^∗∗∗^*p* < 0.001, and ^∗∗∗∗^*p* < 0.0001 vs. the FeSO_4_ group, according to one-way analysis of variance with Tukey's post hoc test.

To investigate HP effects on FeSO_4_-induced neurotoxicity in rat spinal cord neurons, we modeled iron-dependent ROS generation using FeSO_4_ treatment *in vitro*. In Figure 1(a), the experimental conditions of iron-mediated neurotoxicity in primary spinal cord neurons are schematically illustrated. First, we investigated whether HP extracts had any cytotoxic effects in spinal cord neurons. CCK-8 assays confirmed that HP extract concentrations of up to 200 *μ*g/mL did not alter primary spinal cord neuron viability *in vitro* (Figure 1(b)). In FeSO_4_-treated spinal cord neurons, HP extracts at the tested concentrations (10-200 *μ*g/mL) exerted a significant neuroprotective effect against FeSO_4_-induced neurotoxicity (Figure 1(c)). Interestingly, HP increased cell viability in a dose-dependent manner in the treated group, as compared with the FeSO_4_ group.

A live/dead cell assay was additionally performed to determine the viability of cells under identical culture conditions used for the CCK-8 assay. The results revealed a trend similar to that observed in the CCK-8 assay (Figures 1(d) and 1(e)). FeSO_4_-induced neurotoxicity led to a significant reduction in the total number of viable cells (green), as compared to the blank group. In contrast, HP treatment caused changes in the viability of neurons. Spinal cord neurons treated with HP contained a higher number of live cells than dead cells, and the numbers of viable cells increased significantly following HP treatment, in a dose-dependent manner.

We also performed live-cell imaging, which showed that the number of PI-positive neurons in response to FeSO_4_ treatment over 48 h was attenuated by HP treatment (Figure 1(f)). Supplementary Video S1 showed that the axonal growth in response to FeSO_4_ exposure decreased progressively over 48 h, but this was robustly increased by HP treatment.

FeSO_4_-induced cellular toxicity was further examined using Annexin V-FITC/PI double-staining in FACS analysis (Figures 1(g) and 1(h)). Approximately 23.7% of the cells died due to necrosis following FeSO_4_ treatment (Annexin V^−^/PI^+^). In contrast, treatment with HP extract produced gradual decrease in the rate of necrotic cells, which reached a minimum of approximately 14.6%.

Our findings suggest that HP extract can effectively protect neuronal viability under *in vitro* conditions that simulate FeSO_4_-induced oxidative damage. Thus, HP extract can provide neuroprotection against oxidative stress and can be safely applied, as it does not induce neurotoxicity.

### 3.2. HP Promotes NRF2-Related Neurite Outgrowth via Modulation of Iron Homeostasis in FeSO_4_-Treated Primary Spinal Cord Neurons

**Figure 2:**
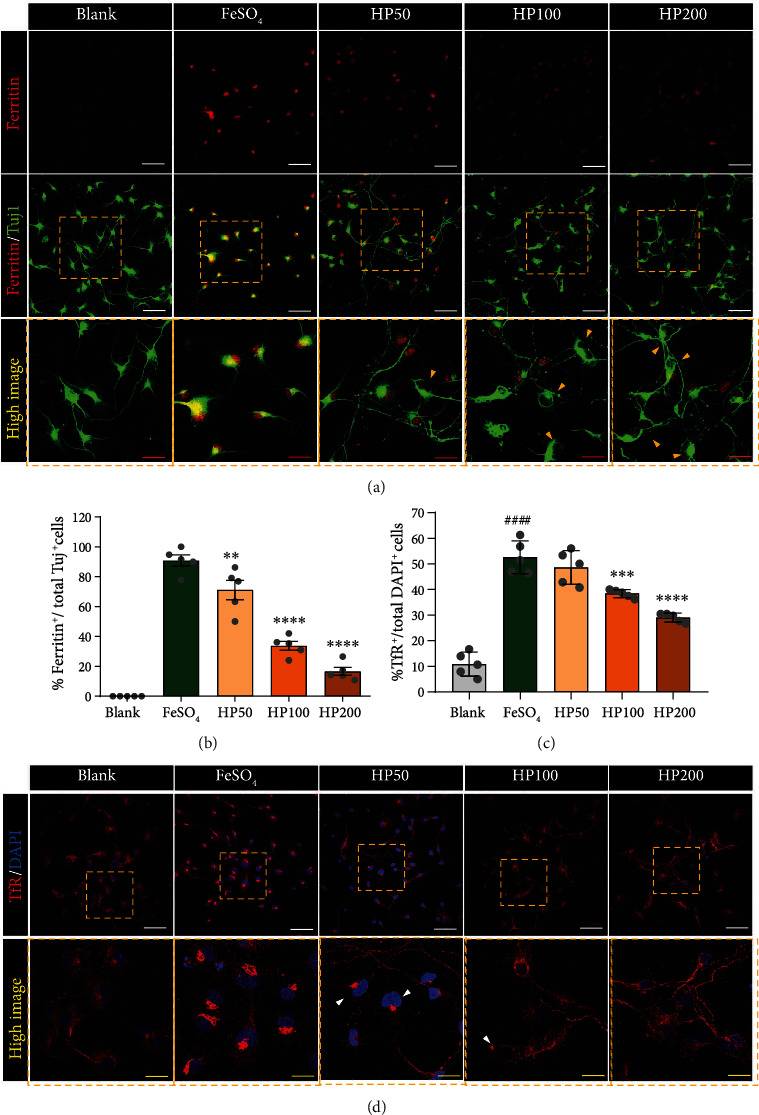
*Harpagophytum procumbens* (HP) regulates intraneuronal accumulation of ferritin via the transferrin receptor. (a) Representative images of ferritin (red)- and Tuj1 (green)-positive neurons in the blank, FeSO_4_, and HP groups. White scale bar = 50 *μ*m; red scale bar = 10 *μ*m. (b) Percentage of cells positive for ferritin in Tuj1-positive neurons. (c) Quantification of the percentage of transferrin-positivity in DAPI-positive neurons after treatment with HP at 50, 100, 200 *μ*g/mL after FeSO_4_-treatment. (d) Representative images of transferrin expression (red) in spinal cord neurons, challenged with HP after FeSO_4_-treatment. White scale bar = 50 *μ*m, yellow scale bar = 10 *μ*m. Data are expressed as means ± SEM. Significant differences are indicated as ^####^*p* < 0.0001 vs. the blank group^,^^∗∗^*p* < 0.01, ^∗∗∗^*p* < 0.001, and ^∗∗∗∗^*p* < 0.0001 vs. the FeSO_4_ group, as analyzed via one-way analysis of variance with Tukey's post hoc test.

Next, we assessed the degree of intracellular iron deposition in spinal cord neurons exposed to FeSO_4_ and evaluated the inhibitory effect of iron accumulation in HP-treated neurons using immunocytochemical ferritin staining (Figure 2(a)). Ferritin can be used to determine how much iron is stored in cells. An abnormally high intracellular ferritin level indicates iron deposition in the cell [30]. In neurons exposed to FeSO_4_, ferritin was strongly expressed in the cell soma and was significantly increased compared to blank group (Figure 2(b)). When various HP concentrations were applied to FeSO_4_-treated neurons, the ferritin expression was substantially decreased in a concentration-dependent manner. We also evaluated the expression of the transferrin receptor (TfR), a known iron carrier transporting iron into cells [31]. FeSO_4_ treatment induced iron accumulation inside spinal cord neurons, which showed enhanced expression of TfR. Meanwhile, the TfR expression observed in the HP-treated cells was significantly and dose-dependently decreased (Figures 2(c) and 2(d)).

**Figure 3:**
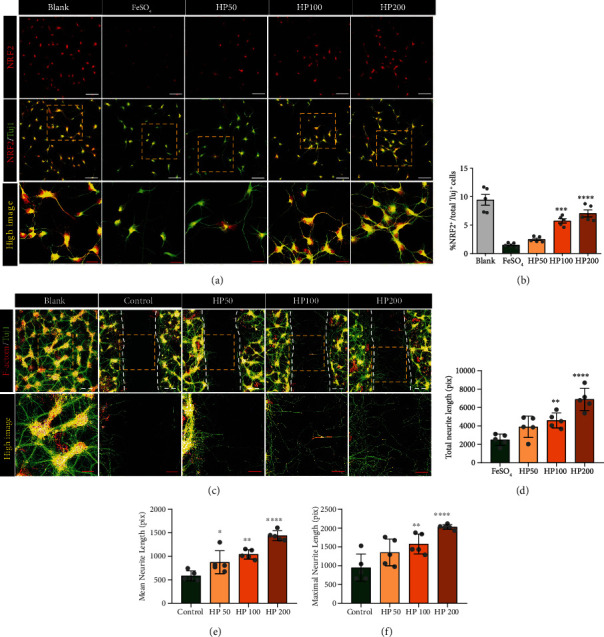
*Harpagophytum procumbens* (HP) exerts NRF2-related neuroprotection effects and promotes neurite outgrowth in FeSO_4_-exposed primary spinal cord neurons. (a) Representative images of NRF2 (red)- and Tuj1 (green)-positive neurons in each group. White scale bar = 50 *μ*m; red scale bar = 10 *μ*m. (b) Percentage of cells positive for NRF2 among Tuj1^+^ neurons. (c) Representative image of the F-actin (red) and Tuj1 (green) stain after laceration injury. White scale bar = 50 *μ*m; red scale bar = 10 *μ*m. (d–f) Quantifying the total, mean, and maximum neurite length within the lacerated area in each group. Data are expressed as the means ± SEM. Significant differences are indicated as ^####^*p* < 0.0001 vs. the blank group, ^∗^*p* < 0.05, ^∗∗^*p* < 0.01, ^∗∗∗^*p* < 0.001, and ^∗∗∗∗^*p* < 0.0001 vs. the FeSO_4_ group were analyzed via one-way analysis of variance with Tukey's post hoc test.

We then investigated whether HP treatment could improve neurite outgrowth in FeSO_4_-treated neurons, by suppressing iron deposition through nuclear factor (erythroid-derived 2)-like 2 (NRF2) upregulation. A previous study had reported that iron homeostasis is modulated by activation of the NRF2 signaling pathway [24]. Immunocytochemical analyses revealed that almost all neurons in the blank group stained positive for NRF2 (Figure 3(a)). In contrast, in neurons exposed to FeSO_4_ for 30 min, the percentage of NRF2 and Tuj1 double-positive neurons were significantly reduced as compared to the blank group (Figure 3(b)). HP treatment increased NRF2 expression in a concentration-dependent pattern.

Furthermore, we compared the lengths of axons following HP treatment in laceration-injured neurons for more intuitive observation of neurite outgrowth (Figure 3(c)). The neurite outgrowths were determined by quantifying the total, mean, and maximum neurite length. These parameters showed significantly longer and dose-dependent effect in the HP groups than in the FeSO_4_ group (Figures 3(d)–3(f)).

These data demonstrated that HP treatment enhances neurite outgrowth concentration-dependently, suggesting that it is a potential neuroprotective agent that modulates iron homeostasis through activation of NRF2 expression in an *in vitro* model of iron deposition.

### 3.3. In Vivo Administration of HP to LSS Rats Reduces Tissue Damage, Inflammatory Response, and Neuropathic Pain

**Figure 4:**
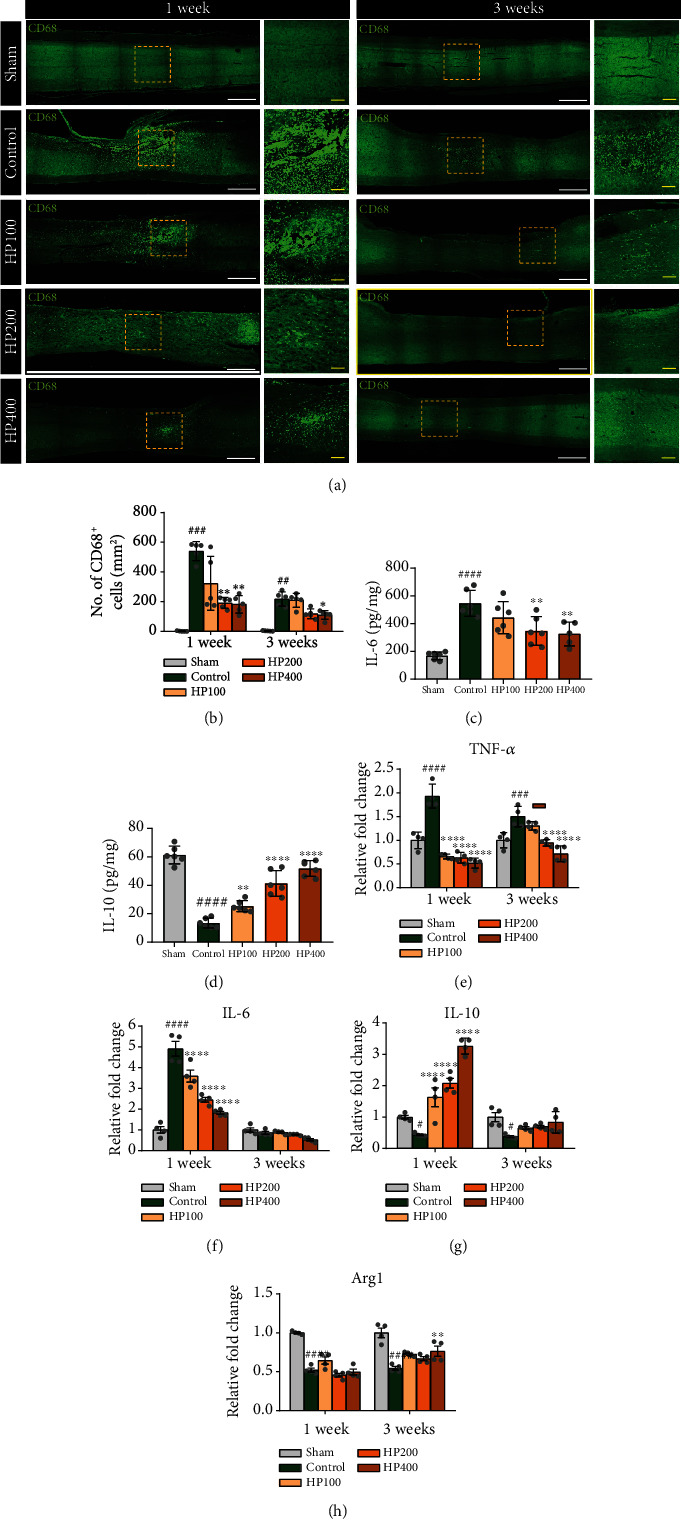
*Harpagophytum procumbens* (HP) attenuates inflammation by regulating M1/M2 status in lumbar spinal stenosis (LSS) model rats. (a) Representative immunohistological images of CD68 (green)-positive macrophages at 1 and 3 weeks after LSS induction and HP administration. White scale bar = 50 *μ*m; red scale bar = 10 *μ*m. (b) Number of cells positive for CD68 in the sagittal spinal cord. (c and d) ELISA analysis of IL-6 and IL-10 at 1 week after LSS and HP administration. (e and f) Proinflammatory-related gene expression analyzed in spinal cord tissue after LSS induction and HP administration in rats, at 1 and 3 weeks; (e) *TNF-a* and (f) *IL-6*. (g and h) Anti-inflammatory-related gene expressions analyzed with spinal cord tissue after LSS and HP administration at 1 and 3 weeks; (g) *IL-10* and (h) *Arg1*. Data are expressed as the means ± SEM. Significant differences are indicated as ^#^*p* < 0.05, ^##^*p* < 0.01, ^###^*p* < 0.001, and ^####^*p* < 0.0001 vs. the sham group, ^∗^*p* < 0.05, ^∗∗^*p* < 0.01, ^∗∗∗^*p* < 0.001, and ^∗∗∗∗^*p* < 0.0001 vs. the control group were analyzed via one-way analysis of variance with Tukey's post hoc test.

H&E staining revealed morphological changes in the spinal cord after intraspinal silicone implantation (Figure. S1). Spinal cord compression was observed in LSS groups with silicone blocks implanted at the L4 level. Inflammation was evaluated based on immunoreactivity of CD68 (Figures 4(a) and 4(b)). The numbers of CD68-positive macrophages were significantly increased within the spinal cord at 1 week after inducing LSS. However, this was substantially decreased after HP administration in a dose-dependent manner.

We also assessed the presence of CD68-positive macrophages at a chronic stage. A relatively lower expression of CD68 was confirmed in each group at 3 weeks than at 1 week in animals with LSS induction. However, the expression pattern at 3 weeks was similar to that at 1 week after LSS induction, and the numbers of macrophages positive for CD68 were significantly lower in the 200 and 400 mg/kg HP groups than in the control group.

We further verified the inhibitory effect of HP administration on the secretion of IL-6, a well-known proinflammatory cytokine, using ELISA (Figure 4(c)). The IL-6 level was significantly higher in the control than in the sham-operated group. However, the levels of IL-6 were significantly decreased following treatment with 200 and 400 mg/kg HP.

In contrast, the expression levels of IL-10, a major anti-inflammatory cytokine that promotes neuron survival and provides trophic support to neurons, were significantly increased in the HP-treated groups, in a dose-dependent manner, at 1 week after LSS induction as compared to the control group (Figure 4(d)).

We also analyzed changes in the expression of anti- and proinflammatory genes at 1 and 3 weeks after LSS induction using qRT-PCR analyses (Figures 4(e)–4(h)). The expression levels of proinflammatory genes, such as *TNF-α* and *IL-6* were significantly upregulated in the control group as compared to the sham-operated group at 1 week, whereas *TNF-α* and *IL-6* levels were significantly downregulated at 1 week in the HP groups as compared to the control group (Figures 4(e) and 4(f)). *TNF-α* expression returned to normal expression ranges in HP-treated groups, dose-dependently, and persisted until 3 weeks. The *IL-6* level was also dose-dependently downregulated at 1 week in the groups administered HP extracts but were not statistically significantly different between groups at 3 weeks. The gene expression of *IL-10*, which encodes an anti-inflammatory cytokine, was downregulated at 1 and 3 weeks in the control group as compared to the sham group, whereas markedly higher levels of *IL-10* gene expression were detected at 1 week in the HP-treated groups. However, the levels were not significantly different among groups at 3 weeks after LSS induction (Figure 4(g)). Arginase 1 is also a marker of anti-inflammatory macrophages. *Arg1* levels were significantly lower in the control group than in the sham group at 1 week. However, we did not observe any differences in *Arg1* levels between control and HP groups. *Arg1* expression showed an increasing tendency at 3 weeks after HP administration, but the difference was only statistically significant in the 400 mg/kg HP group (Figure 4(h)).

**Figure 5:**
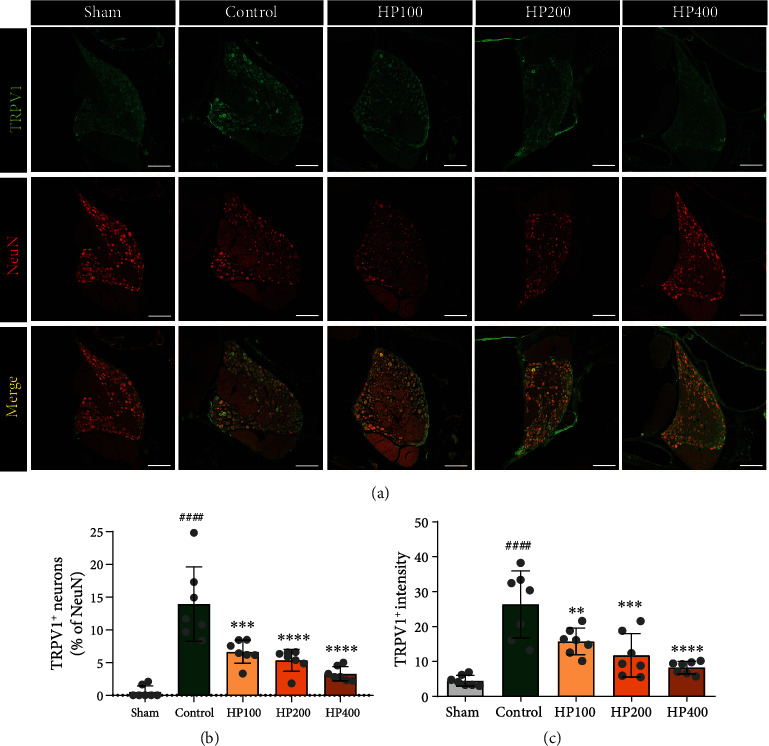
*Harpagophytum procumbens* (HP) prevents upregulation of TRPV1 in dorsal root ganglions (DRG) and attenuates chronic neuropathic pain. (a) Representative immunohistochemical images of TRPV1 (green) and NeuN (red) in DRGs at 3 weeks after LSS in each group. White scale bar = 200 *μ*m. (b) Percentage of TRPV1^+^ neurons that colabeled with NeuN^+^ neurons in DRG. (c) Quantification of fluorescence intensity in TRPV1^+^ neuron within DRG. Data are expressed as the means ± SEM. Significant differences are indicated as ^####^*p* < 0.0001 vs. the sham group, ^∗∗^*p* < 0.01, ^∗∗∗^*p* < 0.001, and ^∗∗∗∗^*p* < 0.0001 vs. the control group, as analyzed by one-way analysis of variance with Tukey's post hoc test.

Next, we examined TRPV1-expressing sensory neurons in the DRG using immunohistochemical staining. TRPV1 expression in DRG neurons is associated with pain pathogenesis in rats with neuropathic pain [32]. Interestingly, LSS substantially upregulated TRPV1 expression in NeuN-positive neurons at 3 weeks, whereas TRPV1 expression in DRG neurons was decreased in HP groups (Figure 5(a)). The percentage of neurons expressing TRPV1 in NeuN^+^ neurons was significantly higher in the control group than in the sham group (Figure 5(b)), while increased TRPV1 expression was dose-dependently inhibited by HP administration in LSS rats. Moreover, TRPV1 intensity showed a trend similar to those observed in the quantification of TRPV1^+^ cells in the DRG (Figure 5(c)).

In summary, our results demonstrated that HP reduces tissue damage, inflammatory cell population, and neuropathic pain in LSS rats.

### 3.4. LSS Induces Alterations of Iron Levels in the Spinal Cord that Correlate with Oxidative Stress

**Figure 6:**
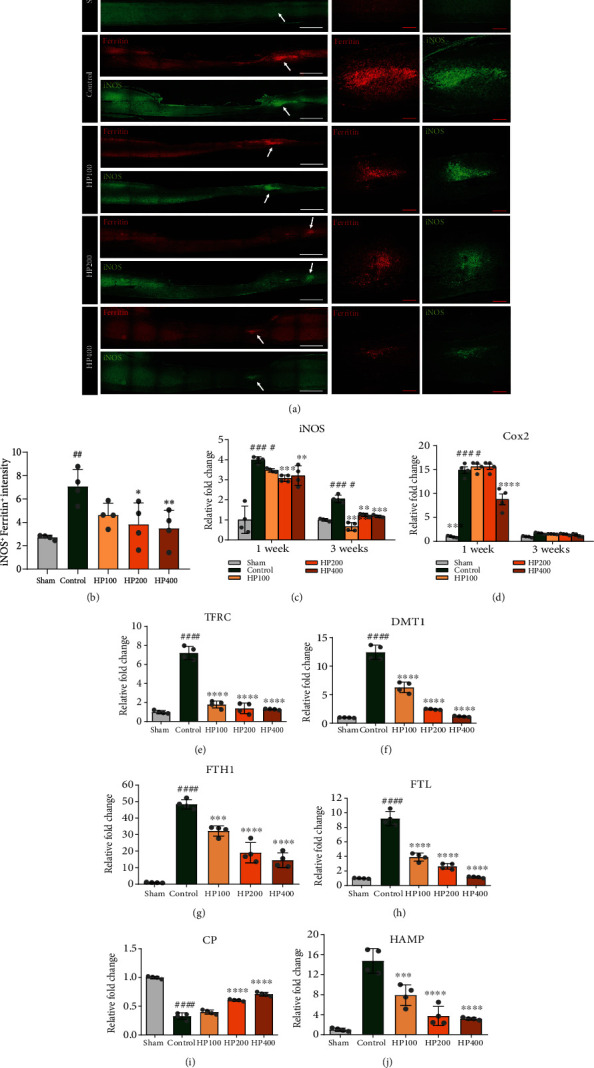
*Harpagophytum procumbens* (HP) ameliorates oxidative stress through attenuating iron deposition after lumbar spinal stenosis (LSS) induction. (a) Representative immunohistochemical images of ferritin (red) and iNOS (green) in spinal cord tissue at 1 week after LSS induction in rats of each group. White scale bar = 1000 *μ*m, red scale bar = 200 *μ*m. (b) Quantitative analysis of iNOS intensity costained with ferritin in spinal cord tissue. (c–j) Gene expression from real-time PCR for oxidative stress-related genes; (c) *iNOS* and (d) *Cox2*, iron metabolism-related genes; (e) *TFRC*, (f) *DMT1*, (g) *FTH1*, (h) *FTL*, (i) *CP*, and (j) *HAMP*. Data are expressed as the means ± SEM. Significant differences are indicated as ^##^*p* < 0.01 and ^####^*p* < 0.0001 compared vs. the sham group, ^∗^*p* < 0.05, ^∗∗^*p* < 0.01, ^∗∗∗^*p* < 0.001, and ^∗∗∗∗^*p* < 0.0001 vs. the control group were analyzed via one-way ANOVA with Tukey's post hoc test.

To evaluate oxidative stress induced by iron deposition in LSS rats and to assess the suppressive effects of HP administration on this oxidative stress, we examined iron level alterations in the spinal cord and their associations with oxidative stress in immunohistochemical colabeling studies using anti-iNOS and antiferritin antibodies. We found that iNOS and ferritin expression was increased after LSS induction, and relatively few double-labeled cells were observed in the HP groups (Figure 6(a)). The average intensity of iNOS costaining with ferritin was significantly increased in the spinal cord of the control group relative to the sham group; however, the intensity dose-dependently decreased in the HP groups (Figure 6(b)). We also performed mRNA analyses of *iNOS* and *Cox2*, which are representative factors of oxidative stress, at 1 and 3 weeks after LSS induction. *iNOS* expression was significantly elevated in the control group as compared to that in the sham group at 1 week, whereas the *iNOS* levels were significantly lower in the 200 and 400 mg/kg HP groups than in the control group. This overall trend was not changed at 3 weeks, although all *iNOS* expression levels at this time point were lower than those at 1 week (Figure 6(c)). Similarly, *Cox2* expression was significantly upregulated at 1 week in the control group as compared to the sham group, but *Cox2* expression was only significantly downregulated in the 400 mg/kg HP group at 1 week. Furthermore, *Cox2* expression was completely downregulated at 3 weeks, with expression levels similar to those of the sham group (Figure 6(d)).

Next, changes in iron metabolism-related genes were confirmed at 1 week after LSS induction, given that changes in iron metabolism are closely associated with LSS-induced oxidative stress. First, we confirmed changes in iron ion transport gene expression after LSS by using real-time PCR analysis (Figures 6(e) and 6(f)). The two main iron influx proteins are transferrin receptor (TFRC) and divalent metal transporter 1 (DMT1). Iron can bind to transferrin (TF), forming transferrin–iron complexes with ferric iron (Fe^3+^) that recognizes the TFRC on the outer cell membrane. The expression level of *TFRC* and *DMT1* were significantly elevated in the control group as compared to the sham group. These genes were significantly and dose-dependently downregulated at 1 week and gradually returned to sham expression ranges after HP treatment. These findings reveal that LSS causes increased iron uptake through TFRC and DMT1 and decreased iron absorption after HP treatment.

We next assessed the ferritin heavy/light chain gene (*FTH1*/*FTL*) expression in each group. Iron uptake and release may be affected by changes in *FTH1*/*FTL* expression. The iron-storage protein ferritin is composed of heavy and light chains. We found that the *FTH1* and *FTL* expression within the spinal cord was significantly higher in the control group than in the sham group at 1 week. In contrast, treating LSS by HP was shown to elicit significant downregulation in the expression of these genes (Figures 6(g) and 6(h)).

We further analyzed the phenomena involved in the excess iron-export mechanism after inducing LSS in rats. Excess iron is exported out of the cell, as ferrous iron (Fe^2+^), through ferroportin (FPN), a transmembrane protein, to maintain cellular iron homeostasis. Iron export by FPN is facilitated by ceruloplasmin (CP) and is blocked by hepcidin. *CP* mRNA expression was significantly downregulated in the control group, whereas it was upregulated at 1 week after HP treatment in LSS rats. In particular, *CP* showed a dose-dependent increase in expression in HP-treated groups (Figure 6(i)).

We also found that expression of *HAMP* was enhanced in the control group but was downregulated significantly in the HP groups in dose-dependent manner (Figure 6(j)). These results indicated that high HAMP levels inhibit iron-export by binding to FPN and induce iron accumulation within the spinal cord in LSS rats. The relatively low expression of *HAMP* after HP treatment was able to induce iron efflux efficiently and maintain iron homeostasis.

Thus, we demonstrated that HP helps maintain iron homeostasis through the regulation of iron metabolism within spinal cords after LSS.

### 3.5. HP Prevents Iron Overload-Induced Oxidative Stress through Activation of NRF2 Signaling in LSS Rats

**Figure 7:**
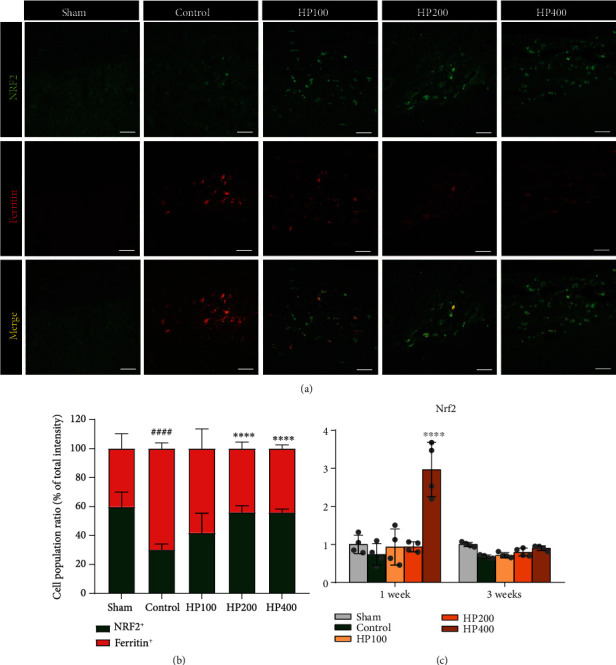
*Harpagophytum procumbens* (HP) prevents iron overload-induced oxidative stress through activation of NRF2 signaling in LSS rats. (a) Representative immunohistochemical images of NRF2 (green) and ferritin (red) in the rat spinal cord at 1 week after LSS induction in each group. (b) The percentage of NRF2^+^ or ferritin^+^ cells to the total intensity within the rat spinal cord in each group. (c) Quantitative *Nrf2* mRNA analysis from real-time PCR at 1 and 3 weeks in each group. Data are expressed as the means ± SEM. Significant differences indicated as ^####^*p* < 0.0001 vs. the sham group, ^∗∗∗∗^*p* < 0.0001 vs. the control group were analyzed via one-way analysis of variance with Tukey's post hoc test.

NRF2-mediated regulation of ferritin, an intercellular iron-storage protein, contributes to iron homeostasis and antioxidative capacity [24]. Therefore, we examined the effects of HP administration on the regulation of ferritin expression via NRF2 in the LSS model. NRF2 expression and its association with ferritin regulation were assessed using immunohistochemical double-staining experiments (anti-NRF2 and antiferritin antibodies; Figure 7(a)). Although NRF2^+^ cells were rarely observed in spinal cord tissue, we confirmed decreased anti-NRF2 staining intensities in ferritin^+^ cells in the control group as compared to those in HP groups. The relative NRF2 and ferritin intensities were measured, and the percentage of the NRF2^+^ or ferritin^+^ intensity to the total intensity was determined (Figure 7(b)). The control group had lower NRF2 intensity than in the sham group, whereas the ferritin intensity was higher in the control group than in the sham group. In contrast, HP groups revealed higher NRF2 and lower ferritin intensities in the spinal cord than those in the control group at 1 week. Furthermore, the HP administration promoted a statistically significant effect on NRF2 expression in 100 and 200 mg/kg. Interestingly, the mRNA level of *Nrf2* in the spinal cord was significantly increased at only 1 week in the 400 mg/kg HP group. The *Nrf2* expression was downregulated in all groups at 3 week and were not significantly different between the groups (Figure 7(c)). Therefore, given that LSS alters NRF2 and ferritin levels in the spinal cord, we looked for potent therapeutic effect of HP administration to enhance NRF2 expression in order to promote an antioxidant effect.

### 3.6. HP Improves Axonal Growth and Functional Recovery in LSS Rats

**Figure 8:**
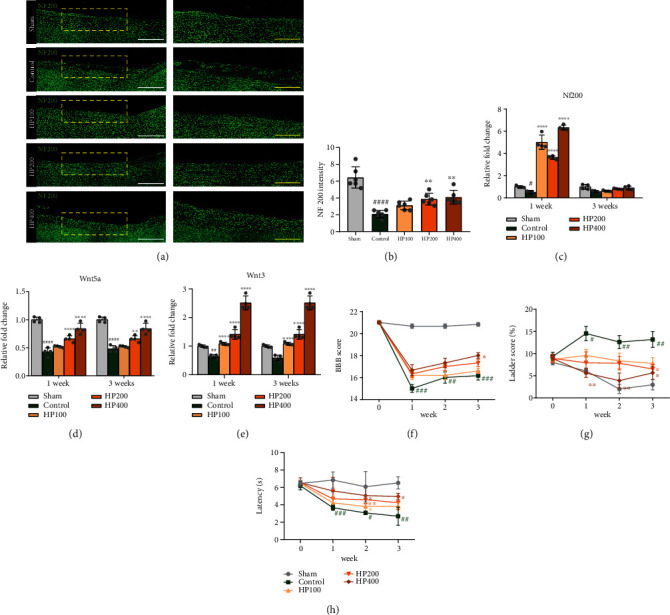
*Harpagophytum procumbens* (HP) protects axonal damage and improves functional recovery in lumbar spinal stenosis (LSS) model rats. (a) Representative immunohistochemical images of NF200 (green) in the rat spinal cord at 3 weeks after LSS induction in rats of each group. (b) The intensity of NF200^+^ axons in each spinal cord, including the implantation site. (c–e) Real-time PCR analysis of mRNA expression levels of regeneration-related genes, (c) *Nf200*, (d) *Wnt5a*, and (e) *Wnt3* at 1 and 3 weeks in each group. (f–h) Locomotor function of LSS rats after application of HP until 3 weeks. (f) BBB score, (g) ladder score, and (h) Von Frey test. Data are expressed as the means ± SEM. Significant differences are indicated as ^#^*p* < 0.05, ^##^*p* < 0.01, ^###^*p* < 0.001, and ^####^*p* < 0.0001 vs. the sham group, ^∗^*p* < 0.05, ^∗∗^*p* < 0.01, ^∗∗∗^*p* < 0.001, and ^∗∗∗∗^*p* < 0.0001 vs. the control group, as analyzed via one-way or two-way analysis of variance with Tukey's post hoc test.

Next, we evaluated the degree of spinal axonal damage using immunohistochemistry of NF200 (an axonal marker) in sagittal sections of the silicon implantation site (Figure 8(a)). Quantification data of the NF200 intensity at the implantation site were higher in the HP groups than in the control group at 3 weeks (Figure 8(b)). There were significant differences among the 200 or 400 mg/kg HP groups and the control group at 3 weeks. Furthermore, several regeneration-related genes, including *Nf200*, *Wnt5a*, and *Wnt3*, were analyzed using real-time PCR at 1 and 3 weeks following LSS induction and HP administration. Interestingly, HP administration of LSS model rats resulted in increased *Nf200* expression in the spinal cord at 1 week. *Nf200* mRNA expression was significantly elevated to a maximum 6-fold increase at 1 week after HP treatment and decreased gradually thereafter, being similar to control levels at 3 weeks (Figure 8(c)). WNT signaling has been regarded as an activator of axon regeneration and transport through the WNT/*β*-catenin (WNT1 and WNT3a) or the WNT/PCP pathways (Wnt5a) [33]. In our LSS model, HP administration induced *Wnt5a* and *Wnt3* upregulation in a dose-dependent manner at 1 and 3 weeks (Figures 8(d) and 8(e)). These findings suggest that HP treatment provides axonal protection and facilitates axonal regeneration after LSS induction.

After HP administration to rats for 3 weeks, the locomotor functions were assessed weekly up to 3 weeks using three methods (BBB, ladder, and Von Frey tests) to determine whether HP treatment is more effective in inducing functional recovery in LSS rats. The HP groups started to show a gradually higher BBB score than the control group from 1 week. The control group had an average BBB score of 16 points at 3 weeks and displayed consistent plantar stepping with consistent forelimb–hindlimb coordination. Toe clearance was also frequently observed during forward limb advancement. The HP groups had an average higher BBB score than the control group at 3 weeks, but the only significant difference was between HP400 group and control group, at 3 weeks, and this was approximately two points higher than the average score of the control group (Figure 8(f)).

Compared with the results of the BBB test, the ladder test revealed more noticeable behavioral differences among the groups. The sham group had a foot fault rate of approximately 10% in 1 week but showed gradual adaptation to ladder test and had a foot fault rate of approximately 3% in the final assessment at 3 weeks. The HP groups showed a decreased foot fault frequency compared to the control group for up to 3 weeks (Figure 8(g)). However, the 400 mg/kg HP group elicited significant but gradual recovery of locomotor function for up to 3 weeks.

In the Von Frey test, the control group showed a withdrawal latency of 4 to 5 seconds in the first week. There was a significant difference between the sham and control groups for up to 3 weeks. Following surgery to induce the LSS model, the rats showed increased sensitivity of the nerves in their legs, resulting in shorter withdrawal latencies. Of note, HP groups showed slower withdrawal latencies than the control group (Figure 8(h)). Therefore, simultaneously administered HP induced locomotor recovery in our LSS model.

## 4. Discussion

In this study, we investigated the effects of HP extract on iron-induced oxidative stress associated with LSS pathophysiology, using *in vitro* and *in vivo* experiments. We demonstrated that HP administration can maintain iron homeostasis within neurons by activating NRF2 signaling, which results in functional recovery of these neurons by regulation of iron-induced oxidative stress.

Studies on oxidative stress in spinal stenosis have reported a significant increase in the expression of 8-OHdG, an indicator of oxidative DNA damage, in LF specimens obtained from patients with spinal stenosis [34]. The findings of previous studies demonstrated that oxidative stress could cause the LF to thicken and harden, which has been suggested to be a major cause of stenosis. However, the route by which an oxidative stress environment causes sufficient to cause nerve compression remained unclear.

We focused on the imbalance of the oxidation/antioxidation system in the LSS, which can lead to oxidative damage. Specifically, for regulation of the antioxidant system, previous studies demonstrated that iron metabolism is a very important mechanism associated with improper functioning of the antioxidation system. Labile iron is a key contributor to oxidative damage in cellular components [35]. Thus, in the present study, we sought to determine changes in iron metabolism in both primary spinal cord neurons and in the spinal cord tissue of rats with LSS. Our *in vitro* analysis revealed that up to 200 *μ*g/mL HP can ameliorate iron neurotoxicity and inhibit iron deposition at primary spinal cord neurons induced by FeSO_4._ Intriguingly, HP was highly effective for promoting neurite outgrowth by activating NRF2 signaling and by regulating the iron uptake through the TFR under FeSO_4_-treated conditions. We also identified excessive iron accumulation in the spinal cord along with an alteration in iron metabolism after LSS induction.

After entry into the mitochondria, iron is utilized for protein synthesis; However, excessive accumulation of iron in cells and tissues facilitates the generation of ROS and destroys redox homeostasis through the Fenton and Haber–Weiss reactions [36]. Therefore, increased iron levels can cause oxidative damage to biomolecules, such as intracellular proteins, lipids, and DNA, and could lead to increased oxidative stress. Recently, ferroptosis, an iron-dependent form of nonapoptotic cell death, has emerged as a major cause of neuronal cell death in various neurological disorders, by inducing iron-dependent ROS generation and accumulation of peroxidized lipids [37, 38]. Our results suggest a novel pathophysiological mechanism for the etiology of spinal stenosis, involving the activation of oxidative factors and creation of an oxidative stress environment induced by iron accumulation. Furthermore, we confirmed the ability of the HP extract to promote functional recovery from spinal stenosis and to inhibit oxidative damage by maintaining iron homeostasis through the activation of NRF2 and effective inhibition of iron accumulation in neurons.

However, in the spinal stenosis animal model that we established, LF hypertrophy was not induced directly; rather, artificial nerve compression was created by grafting silicon on top of the spinal LF. Therefore, studies investigating the direct association between oxidative stress and iron accumulation in hypertrophic LF tissues are still lacking. Future studies should focus on verifying the fundamental effects and mechanism of action of HP extract in terms of iron homeostasis and oxidative stress in hypertrophic LF specimens. This may be achieved by identifying changes in iron metabolism after treating LF cells with oxidative stress factors *in vitro*, along with the development of a spinal stenosis animal model with directly induced LF hypertrophy.

Furthermore, another limitation of our study was that it was difficult to identify a single target responsible for the iron homeostasis mechanism of the HP extract, which is a complex multiherbal formula. Previous studies have reported that the main active components found in HP extract are harpagoside, harpagide, 8-coumaroylharpagide, and verbascoside [39]. Among these, harpagoside, belonging to the iridoid glycoside family, was considered the major active component with antioxidant activity. Current evidence reported that harpagoside inhibited lipopolysaccharide-induced cyclooxygenase-2 activity and inducible nitric oxide expression through the suppression of NF-*κ*B activation in HepG2 cells [40]. In another study, harpagoside was considered the main cause of the antioxidant properties of HP extract, as it inhibited nitric oxide production [41, 42]. Further studies are needed to investigate the iron homeostasis-related effect of harpagoside in an LSS model.

## 5. Conclusions

Our findings indicate that LSS is associated both with a dysregulation of iron metabolism and an inability to maintain iron homeostasis. Moreover, the effect of HP against oxidative damage through excess iron accumulation in the spinal cord were confirmed. Our results suggest that the HP extract improved functional recovery after LSS by promoting antioxidative defenses through NRF2-mediated iron signaling changes. Furthermore, our results provide a resource for determining a standard treatment direction for oxidative stress regulation in the treatment of LSS patients.

## Figures and Tables

**Table 1:** Primer sequences used for real-time polymerase chain reaction analysis.

Gene	5′–3′	Primer sequence
*TNF-α*	Forward	CCGACTACGTGCTCCTCACC
	Reverse	CTCCAAAGTAGACCTGCCCG
*IL-6*	Forward	CCACCCACAACAGACCAGTA
	Reverse	GGAACTCCAGAAGACCAGAGC
*IL-1β*	Forward	TTGCTTCCAAGCCCTTGACT
	Reverse	GGTCGTCATCATCCCACGAG
*IL-10*	Forward	TAACTGCACCCACTTCCCAG
	Reverse	AGGCTTGGCAACCCAAGTAA
*Arg1*	Forward	GTCTCCAGATGCCTTTGCTTC
	Reverse	ATGAAATTCAGGGTGTGGGAAT
*iNOS*	Forward	ATGGCTTGCCCCTGGAAGTT
	Reverse	TGTTGGGCTGGGAATAGCAC
*Cox2*	Forward	CTCAGCCATGCAGCAAATCC
	Reverse	GGGTGGGCTTCAGCAGTAAT
*TFRC*	Forward	GGCTATGAGGAACCAGACCGCTACA
	Reverse	TGGACTTCGCAACACCAGGGC
*DMT1*	Forward	TGTCGCCTGTCCATTTGGCCG
	Reverse	TGGCGTGGCGGGGTTGAAAT
*Fth1*	Forward	TTGCAACTTCGTCGCTCCGCC
	Reverse	TGGCGCACTTGCGAGGGAGA
*Nrf2*	Forward	GATCTGTCAGCTACTCCCAG
	Reverse	GCAAGCGACTCATGGTCATC
*Nf200*	Forward	AACACCACTTAGATGGCGGG
	Reverse	ACGTGGAGCGTTCAGCAATA
*Wnt3*	Forward	CCAATTTGGTGGTCCCTGGC
	Reverse	TAATTGCGGCAGAAACGCAG
*Wnt5a*	Forward	GTTGAAGCCACAAGAGACAGC
	Reverse	AGAGCATGAGCCTTTTCGGT
*Gapdh*	Forward	CCCCCAATGTATCCGTTGTG
	Reverse	TAGCCCAGGATGCCCTTTAGT

## Data Availability

No data were used to support this study.

## Supplementary Materials

Supplementary Materials Figure S1: representative H&E images of spinal cords for each sagittal section after LSS induction. Video S1: live cell imaging of PI-positive dying cells in the spinal cord neurons. (https://www.dropbox.com/s/jm9eb4710x28xo9/Supplimentary%20Video%201.wmv?dl=0).
