# Supplementary material for: Harpagophytum procumbens Inhibits Iron Overload-Induced Oxidative Stress through Activation of Nrf2 Signaling in a Rat Model of Lumbar Spinal Stenosis
Source: Oxid Med Cell Longev. 2022 Sep 14;2022:3472443. doi: 10.1155/2022/3472443 (PMC9492433; doi:10.1155/2022/3472443)
Supplement: Supplementary Materials — Figure S1: representative H&E images of spinal cords for each sagittal section after LSS induction. Video S1: live cell imaging of PI-positive dying cells in the spinal cord neurons. (https://www.dropbox.com/s/jm9eb4710x28xo9/Supplimentary%20Video%201.wmv?dl=0). [file 3472443.f1.docx]

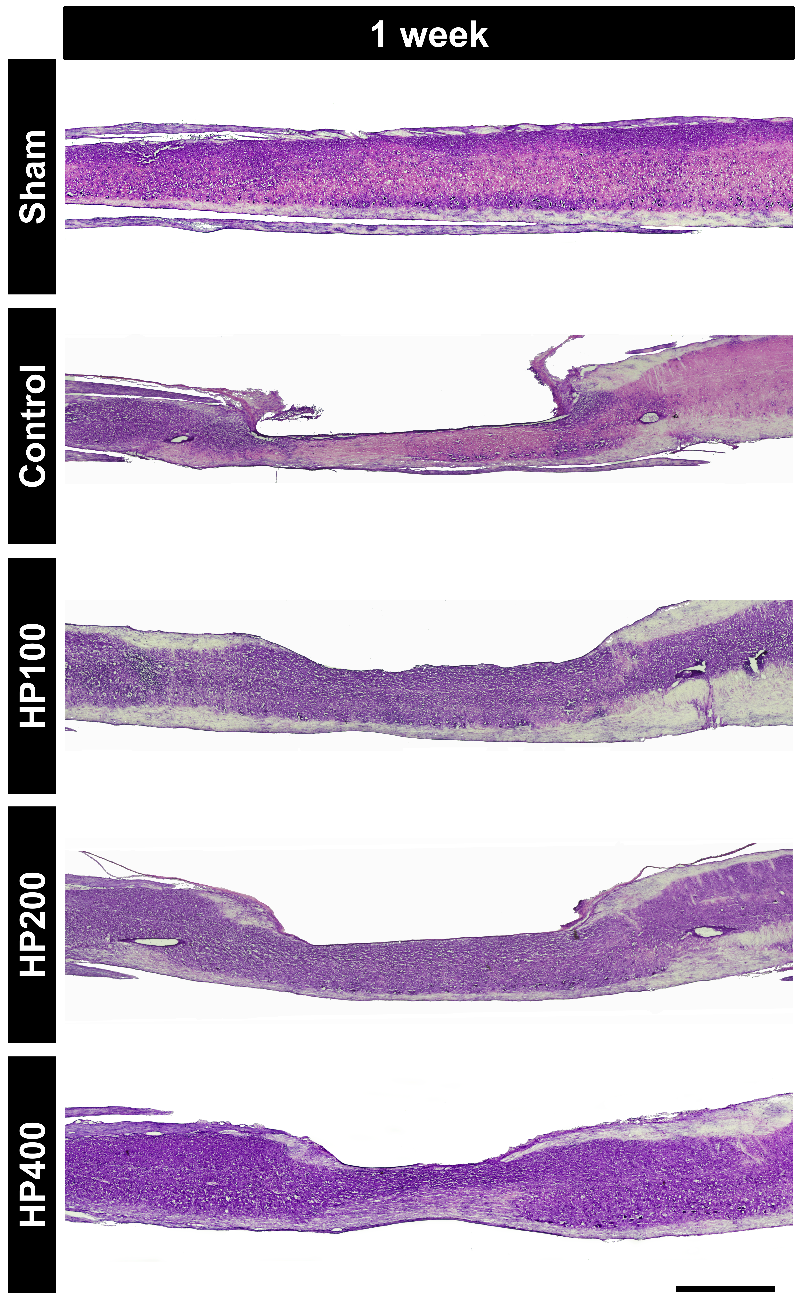


**Figure S1.** Representative H&E images of spinal cords in each group after LSS induction. Black scale bar = 2 mm.
